# The natural product salicin alleviates osteoarthritis progression by binding to IRE1α and inhibiting endoplasmic reticulum stress through the IRE1α-IκBα-p65 signaling pathway

**DOI:** 10.1038/s12276-022-00879-w

**Published:** 2022-11-10

**Authors:** Zhenglin Zhu, Shengqiang Gao, Cheng Chen, Wei Xu, Pengcheng Xiao, Zhiyu Chen, Chengcheng Du, Bowen Chen, Yan Gao, Chunli Wang, Junyi Liao, Wei Huang

**Affiliations:** 1 Department of Orthopaedic Surgery, The First Affiliated Hospital of Chongqing Medical University, 400016 Chongqing, China; 2 Orthopaedic Research Laboratory, Chongqing Medical University, 400016 Chongqing, China; 3 National Innovation and Attracting Talents “111” base, Key Laboratory of Biorheological Science and Technology, Ministry of Education, College of Bioengineering, Chongqing University, 400030 Chongqing, China

**Keywords:** Osteoarthritis, Molecularly targeted therapy, Single-molecule biophysics, Drug development

## Abstract

Despite the high prevalence of osteoarthritis (OA) in older populations, disease-modifying OA drugs (DMOADs) are still lacking. This study was performed to investigate the effects and mechanisms of the small molecular drug salicin (SA) on OA progression. Primary rat chondrocytes were stimulated with TNF-α and treated with or without SA. Inflammatory factors, cartilage matrix degeneration markers, and cell proliferation and apoptosis markers were detected at the mRNA and protein levels. Cell proliferation and apoptosis were evaluated by EdU assays or flow cytometric analysis. RNA sequencing, molecular docking and drug affinity-responsive target stability analyses were used to clarify the mechanisms. The rat OA model was used to evaluate the effect of intra-articular injection of SA on OA progression. We found that SA rescued TNF-α-induced degeneration of the cartilage matrix, inhibition of chondrocyte proliferation, and promotion of chondrocyte apoptosis. Mechanistically, SA directly binds to IRE1α and occupies the IRE1α phosphorylation site, preventing IRE1α phosphorylation and regulating IRE1α-mediated endoplasmic reticulum (ER) stress by IRE1α-IκBα-p65 signaling. Finally, intra-articular injection of SA-loaded lactic-co-glycolic acid (PLGA) ameliorated OA progression by inhibiting IRE1α-mediated ER stress in the OA model. In conclusion, SA alleviates OA by directly binding to the ER stress regulator IRE1α and inhibits IRE1α-mediated ER stress via IRE1α-IκBα-p65 signaling. Topical use of the small molecular drug SA shows potential to modify OA progression.

## Osteoarthritis: Natural bark extract could limit disease progression

Salicin, a small molecule extracted from willow bark, could provide a safe, effective injectable treatment for osteoarthritis, particularly in the early stages of the disease. Osteoarthritis is driven by stress in the intracellular endoplasmic reticulum (ER), which results in chronic inflammation and death of chondrocytes, the cells involved in cartilage formation. Salicin, which is metabolized into salicylic acid after oral ingestion, already forms the basis of aspirin and other anti-inflammatory pain relievers, but the exact mechanisms of its action in osteoarthritis are unclear. In experiments on cell cultures and rats, Junyi Liao, Wei Huang and co-workers at Chongqing Medical University, Chongqing, China, showed how salicin binds directly to a protein involved in promoting ER stress, blocking its activity. This in turn prevented the degeneration of the cartilage matrix and boosted levels of healthy chondrocytes.

## Introduction

Osteoarthritis (OA) is the most prevalent joint disease and affects an estimated 500 million people worldwide^1^. Identification of disease-modifying OA drugs (DMOADs) that modify the structural progression of OA is considered a potential strategy for the treatment of early- or middle-stage OA^2,3^.

Willow bark is regarded as a successful example of a modern drug developed from an herbal remedy, which was first described ~200 years ago. Salicin (SA) is the main chemically standardized willow bark extract; its chemical oxidation resulted in a new substance termed “salicylic acid”, and the acetylated derivative was finally turned into the famous drug called “aspirin”^4,5^. SA is metabolized into salicylic acid after oral ingestion and plays roles in the treatment of pain, headaches, and inflammatory conditions^5,6^. However, the formation of salicylic acid alone is unlikely to explain the analgesic or anti-rheumatic effects of willow bark^7^, which indicates potential mechanisms that need further clarification. In contrast, as a small molecule drug, SA is easy to manufacture and can be easily absorbed and directly cross cell membranes. Recent studies have shown that SA helps prevent inflammation and angiogenic effects^8^, prevents cellular senescence^9^, and exhibits anti-irritation and anti-aging effects in dermatological applications^10^. However, based on our research, although SA appears efficient and safe to use in the treatment of OA^7,11–13^, the mechanisms by which SA prevents OA cartilage degeneration have not been fully elucidated.

In this study, we investigated the effects of SA on cartilage degeneration by both in vitro and in vivo tests. We clarified that SA prevents cartilage degeneration by alleviating inositol-requiring enzyme 1α (IRE1α) signaling-mediated endoplasmic reticulum (ER) stress. Our findings indicated the potential therapeutic effects of SA on OA progression by intra-articular injection.

## Materials and methods

### Chondrocyte culture and chemicals

The experimental protocols were approved by the Institutional Review Board (IRB) of Chongqing Medical University (No. 2020-018). Male Sprague‒Dawley (SD) rats were fed in specific pathogen-free animal facilities. Primary rat knee chondrocytes were isolated from articular cartilage of 4-day-old neonatal rats, as described previously^14^. Chondrocytes at passage 0 or 1 were subjected to the following experiments.

The purity of SA (Selleck Chemicals, TX, USA) was 98.9523% according to the manufacturer’s examination report with the use of high-performance liquid chromatography (HPLC). SA was dissolved in 0.1% dimethylsulfoxide (DMSO), and 0.1% DMSO was used as the control. Tumor necrosis factor α (TNF-α) was purchased from Peprotech (NJ, USA), and APY29 was purchased from Selleck Chemicals (TX, USA) at a concentration of 10 μM, as previously described^15,16^. Unless indicated otherwise, all chemicals were purchased from Sigma-Aldrich or Corning.

### Cell viability

Cell viability was determined by Cell Counting Kit-8 (CCK-8, Med Chem Express, Shanghai, China) assays following the manufacturer’s protocol. For identification of toxic dosages, chondrocytes were treated with SA or TNF-α in gradient concentrations (0–100 μM for SA and 0–200 ng/ml for TNF-α) for 48 h. For the sustained toxicity of SA or TNF-α, chondrocytes were cultured in medium containing SA at different concentrations for 5 days and TNF-α for 3 days. The optical density (OD) values were determined at a wavelength of 450 nm. Nonlinear regression analysis was used to calculate the half-maximal inhibitory concentration (IC50) values for SA (percent cell proliferation versus concentration). The concentration of TNF-α in the present study was 10 ng/ml for 48 h according to previous studies^9,17–21^. Cytotoxicity of TNF-α on primary articular chondrocytes was shown in Supplementary Fig. 1.

### Reverse transcription-quantitative polymerase chain reaction and XBP1 mRNA splicing

Total RNA was extracted from the chondrocytes with TRIzol reagent (Thermo Fisher Scientific, MA, USA) according to the manufacturer’s instructions. Reverse transcription was carried out by using the EvoScript Universal cDNA Master Reagent Kit (Med Chem Express, Shanghai, China). SYBR Green qPCR Master Mix (Med Chem Express, Shanghai, China), cDNA and primers were mixed according to the manufacturer’s instructions. The qPCR protocols were as follows: 5 min at 95 °C, 40 cycles of 10 s at 95 °C, 20 s at the optimal temperature for each pair of primers, and 20 s at 72 °C. Melting curves were generated at every endpoint of amplification for 10 s at 95 °C before 30 increments of 0.5 °C from 65 to 95 °C. GAPDH was used as the reference gene. All sample values were normalized to GAPDH expression by using the 2^−△△CT^ method. All qPCRs were performed with three independent experiments. The qPCR primer sequences are shown in Table 1.

**Table 1:** List of reverse transcription-quantitative polymerase chain reaction primers.

Genes	Primer sequences (forward/reverse)
rat IL1-β	5ʹ-TGACTTCACCATGGAACCCG-3ʹ/5ʹ-GACCTGACTTGGCAGAGGAC -3ʹ
rat IL-6	5ʹ-CTTCACAGAGGATACCACCCACA − 3ʹ/5ʹ-AATCAGAATTGCCATTGCACAAC-3ʹ
rat MMP13	5ʹ-ACCCAGCCCTATCCCTTGAT -3ʹ/5ʹ- TCTCGGGATGGATGCTCGTA -3ʹ
rat COL2A1	5ʹ-AATTTGGTGTGGACATAGGG -3ʹ/5ʹ- AAGTATTTGGGTCCTTTGGG -3ʹ
rat ACAN	5ʹ-AACTTCTTCGGAGTGGGTGGT -3ʹ/5ʹ- CAGGCTCTGAGACAGTGGGG -3ʹ
rat BCL2	5ʹ-GGTGGGGTCATGTGTGTGG -3ʹ/5ʹ- CAGCGGTAGGTGTCGAAGC -3ʹ
rat CDK1	5ʹ-TCCTCCAGGGGATTGTGTTTT -3ʹ/5ʹ- GCCAGTTTGATTGTTCCTTTGTC -3ʹ
rat MKI67	5ʹ-GCCCCTGGAAGATTATGGTGG -3ʹ/5ʹ- GGGTTCTGACTGGTTGTGGTTGT -3ʹ
rat GAPDH	5ʹ-TCTCGGGATGGATGCTCGTA -3ʹ/5ʹ- CAGATCCACAACGGATACAT -3ʹ

XBP1 mRNA splicing was assessed with reverse transcription polymerase chain reaction (RT‒PCR). Total RNA was purified using an RNeasy Mini Kit (Qiagen) and then converted into cDNA by using an EvoScript Universal cDNA Master Reagent Kit (Med Chem Express, Shanghai, China) following the manufacturer’s protocols. cDNA samples were diluted (1:10) and amplified using the primers F: 5ʹ- TTACGAGAGAAAACTCATGGC-3ʹ and R: 5ʹ-GGGTCCAAGTTGTCCAGAATGC-3ʹ with RT‒PCR and assessed on 2.5% agarose gels as previously reported^22^.

### Western blotting

Chondrocytes treated with or without SA were lysed in cold radioimmunoprecipitation (RIPA, Beyotime, Shanghai, China) buffer containing phosphatase and protease inhibitors and then centrifuged at 13,000 × *g* at 4 °C for 15 min. Equivalent quantities of protein (30 μg) in each group were separated by 10–12% SDS-polyacrylamide gel electrophoresis (SDS‒PAGE) and transferred to a polyvinylidene difluoride (PVDF) membrane (Roche, Basel, Switzerland). For the detection of aggrecan, samples were deglycosylated according to the manufacturer’s instructions. After the membranes were blocked with 5% skim milk, they were probed with primary antibodies against Col2α1 (Abcam), Aggrecan (Abcam), MMP13 (Zen Bio), B-cell lymphoma 2 (BCL2) (Zen Bio), Cyclin Dependent Kinase 1 (CDK1) (Abcam), total extracellular signal-regulated kinase 1/2 (ERK1/2) (CST), phospho-ERK1/2 (CST), total-IκBα (CST), phospho-IκBα (CST), phospho-IRE1α (Zen Bio), IRE1α (Bioss), GRP78 (Zen Bio), NF-κB p65 (CST), phospho-NF-κB p65 (CST), phospho-PERK (Thr982) (Zen Bio), PERK (Zen Bio), ATF6 (ABclonal), XBP-1s (CST), and GAPDH (Zen Bio) overnight at 4 °C. Horseradish peroxidase-conjugated anti-mouse or anti-rabbit IgG (Abcam) was used as the secondary antibody. An enhanced chemiluminescence (ECL, Thermo Fisher Scientific, MA, USA) detection system was used to detect the protein bands on the membrane. The intensity of the protein bands was analyzed by ImageJ software using GAPDH as a reference protein as previously described^23,24^.

### Cell immunofluorescence assays

Cells on coverslips were incubated with 6% normal goat serum and 4% bovine serum albumin in phosphate-buffered saline (PBS) for 1 h at 37 °C. Then, the cells were incubated with NF-κB p65 antibody (CST) and phospho-NF-κB p65 antibody (CST) overnight at 4 °C. The following day, fluorescein isothiocyanate (FITC/CY3)-labeled goat anti-rabbit IgG was used as a secondary antibody, and the cells were incubated for 1 h at room temperature. Finally, the cells were counterstained with 4′,6-diamidino-2-phenylindole (DAPI) and observed by fluorescence microscopy.

### Cell proliferation assay

Chondrocyte proliferation was quantified by an ethynyl deoxyuridine (EdU) DNA in vitro proliferation detection kit (RiboBio, Guangzhou, China) according to the manufacturer’s instructions. The number of EdU-labeled cells was calculated from fields randomly selected in each well by two independent laboratory technicians in a blinded, random fashion.

### Cell apoptosis assay

Chondrocytes in each treatment group were trypsinized, collected and washed three times with cold PBS. Approximately 4 × 10^5^ cells/ml suspensions per group were mixed with Annexin V-FITC and PI binding buffer for 20 min according to the standard protocol of the Annexin V-FITC kit (Beyotime, Beijing, China). Finally, the mixtures were subjected to a flow cytometer (BD Biosciences, CA, USA) for apoptosis analysis.

### ELISA

The levels of MMP13 in chondrocyte supernatants were measured using ELISA kits (E-EL-R0045c, Elabscience, Wuhan, China) according to the manufacturer’s instructions. The OD value was recorded at a wavelength of 450 nm.

### RNA-sequencing and bioinformatics analysis

Total RNA extracted from chondrocytes treated with or without SA was subjected to RNA-sequencing (RNA-Seq). RNA samples were sent to OE Biomedical Technology Co., Ltd., (Shanghai, China) for RNA-Seq. RNA sequencing was performed on the HiSeq2500 system (Illumina, CA, USA). The R programming language program was used to analyze the raw data and identify differentially expressed genes (DEGs). Selection of differentially expressed transcripts with *p* values ≤ 0.05 and fold change ≥2 was followed by Gene Ontology (GO) biological function enrichment analysis and Kyoto Encyclopedia of Genes and Genomes (KEGG) signal pathway enrichment analysis.

### Target prediction and molecular docking

The SA structure was downloaded from the PubChem database and converted to mol2 format. The three-dimensional structure of the IRE1α (PDB ID: 6HX1) protein was downloaded from the Research Collaboratory for Structural Bioinformatics (RCSB) protein database. AutoDock Vina 1.1.2 was used for semiflexible docking with SA and IRE1α. The parameter exhaustiveness was set as 20 to increase the calculation accuracy. The conformation with the best affinity (-8.567 kcal/mol) was selected as the final docking conformation.

### Drug affinity responsive target stability

Drug affinity responsive target stability (DARTS) was performed as previously described^25,26^. Briefly, chondrocytes were treated with cold M-PER lysis buffer (Thermo Fisher, MA, USA) containing a protease inhibitor cocktail (1 mM Na3VO4 and 1 mM NaF). Protein lysates were first mixed with 10x TNC buffer (500 mM Tris–HCl pH=8.0, 500 mM NaCl and 100 mM CaCl2 at a ratio of 1:1) and then incubated with DMSO or SA for 1 h at room temperature. Next, the sample was proteolyzed in gradient concentrations of pronase (Roche, Basel, Switzerland) for 10 min, which was followed by the addition of 2 μl of cold 20x protease inhibitor cocktail. Coomassie blue staining (Beyotime, Shanghai, China) was carried out to estimate the relative abundance of proteins. An equal portion of each sample was loaded onto an SDS‒PAGE gel for Western blotting.

### Animal model

All animal experiments were approved by the Institutional Review Board (IRB) of Chongqing Medical University (No. 2020-018). After anesthesia and standard aseptic surgical procedures, ACLT was performed in the right knee of rats (200 ± 20 g) to establish an OA model (duration: four weeks)^20,21^, and rats with only open knee joints were regarded as a sham group (*n* = 5). Poly(lactic-co-glycolic acid) (PLGA) (Sigma-Aldrich, MO, USA) was dissolved in CH_2_Cl_2_ and mixed with ultrasonically dissolved SA, and then, PLGA-loaded SA was obtained by the double-emulsion method as reported previously^27,28^. The animals that received ACLT were randomly divided into three groups: ACLT (PBS only, *n* = 5), vehicle (PLGA vehicle, *n* = 5), and SA (PLGA vehicle with SA, *n* = 5). Four weeks after surgery, intra-articular injection of 100 μL of PLGA + SA suspension (the equivalent of 1 mM SA, PLGA loaded), PLGA only and PBS only was performed. Four weeks post-intra-articular injection, the rats were sacrificed, and knee joints were harvested.

### Histological analysis

Samples were fixed in 4% paraformaldehyde (Beyotime, Beijing, China), decalcified in 0.5 M ethylenediaminetetraacetic acid (EDTA) and embedded in paraffin. Serial 5-μm-thick sections were obtained and subjected to histological and other specialty staining evaluations, including hematoxylin-eosin (H&E) and safranin O/green staining, as previously described^22,24,29^. The articular surface of the femur and tibia in coronal sections of the knee joint was qualitatively and semiquantitatively analyzed as previously described^20,21^. Each specimen was scored by three orthopedic surgeons following the double-blind principle according to the Osteoarthritis Research Society International (OARSI) criteria for rats^30^.

For tissue IF staining, paraffin sections were dehydrated in ethanol solutions of different concentrations, permeabilized with 0.1% Triton X-100 and blocked with 5% BSA. Then, the sections were incubated overnight at 4 °C with primary phospho-NF-κB p65 (CST), Ki-67 (D3B5) (CST) and p-IRE1α (Zen Bio) antibodies. Sections were treated with appropriate fluorescein-conjugated secondary antibodies. DAPI was utilized for nuclear staining. The TUNEL assay was carried out using a Click-iTTM Plus TUNEL Apoptosis Assay Kit (Invitrogen) according to the manufacturer’s instructions. The images of positive cells were captured by a fluorescence microscope.

### Sample size determinations

For in vitro experiments with fold change or quantitative analysis, we calculated a minimum sample size (*n* ≥ 3) with three independent experiments. For in vivo experiments, based on previous experiments, we selected a sample size of *n* ≥ 4 at an alpha level of 0.05 and a power of 80%^31,32^.

### Statistical analysis

All datasets were compared using GraphPad Prism (GraphPad Software, La Jolla, CA) version 9.0. Data are shown as the mean ± standard deviation (SD). For in vivo and vitro studies, unpaired Student’s *t* test (for two groups) and one-way ANOVA (for multiple groups) were used followed by the Tukey‒Kramer test. *p* < 0.05 was considered statistically significant.

## Results

### Phenotypic characterization of primary articular chondrocytes and the cytotoxicity of SA on chondrocytes

**Fig. 1:**
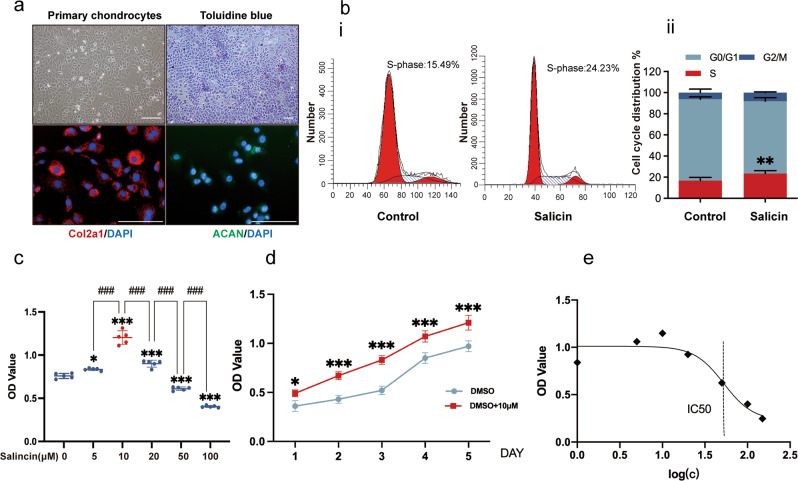
Phenotypic characterization of primary articular chondrocytes and the cytotoxicity of SA on chondrocytes. **a** Staining of articular chondrocytes in primary cultures with toluidine blue. Immunostaining of primary articular chondrocytes with Col2a1 (red), ACAN (green) and DAPI (blue). **b** Flow cytometry for cell cycle progression of chondrocytes with or without SA (i). Quantitative analysis (ii) found that SA significantly promoted S-phase cells (*n* = 3, Student’s t test). **c** Chondrocytes were treated with SA at gradient concentrations (0-100 μM) for 48 h and subjected to CCK-8 analysis. From 5 μM to 20 μM SA treatment, the OD values were significantly higher than those of the 0 μM (DMSO) group, and for 50 μM and 100 μM SA treatment, the OD values were significantly lower than those of the 0 μM (DMSO) group (*n* = 5, one-way ANOVA). **d** Chondrocyte proliferation curve with SA treatment from Day 1 to Day 5. Compared with DMSO treatment, 10 μM SA treatment significantly promoted cell proliferation from Day 1 to Day 5 (*n* = 5, Student’s *t* test). **e** IC50 of SA on chondrocytes; concentrations were transferred to Log(c). The data are expressed as the mean ± SD, *p < 0.05, **p < 0.01, ***p < 0.001, *compared with the DMSO group, #*p* < 0.05, ##*p* < 0.01, ###*p* < 0.001, # compared with the indicated group.

Primary articular chondrocytes were characterized by toluidine blue staining and IF staining with Col2a1 (red) and ACAN (green) (Fig. 1a). We first identified the effects of SA on the cell cycle and found that SA promoted chondrocyte proliferation (Fig. 1bi). Specifically, quantitative analysis (Fig. 1bii) showed that SA significantly increased S-phase chondrocytes. Next, the cytotoxicity of SA to chondrocytes was assessed. As shown in Fig. 1c, 5 μM, 10 μM and 20 μM SA resulted in significant effects, and 10 μM had the strongest effect. However, 50 μM and 100 μM SA dramatically reduced cell viability. Furthermore, we found that 10 μM SA treatment promoted chondrocyte proliferation from Day 1 to Day 4 compared with the control treatment (Fig. 1d). For these experiments, the IC50 of SA was 42.30 μM (Fig. 1e). Thus, 10 μM SA was considered not toxic to chondrocytes and selected as the optimal dose for the next investigations.

### SA inhibits TNF-α-induced chondrocyte inflammatory factor expression and extracellular matrix degeneration

**Fig. 2:**
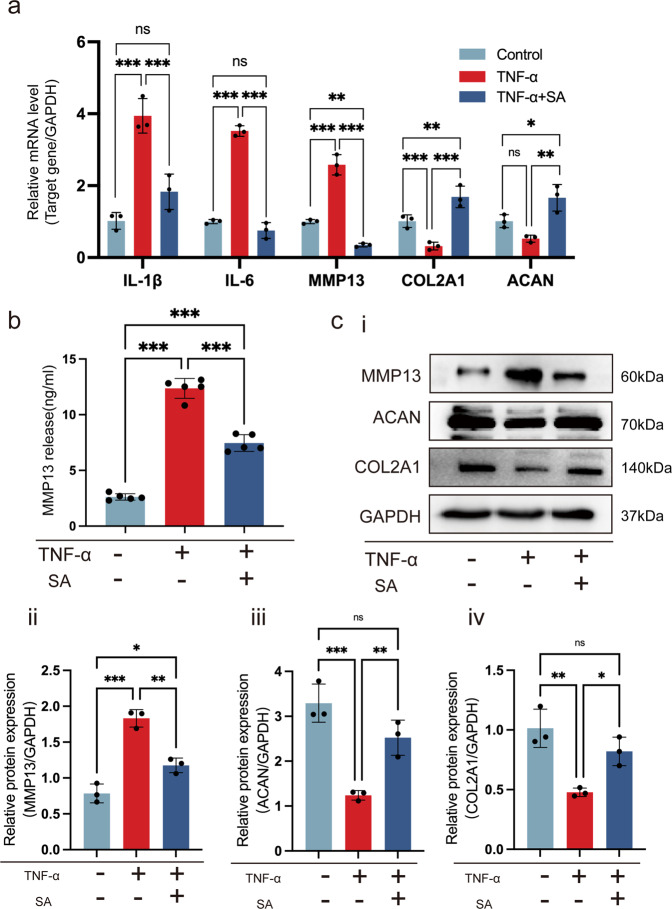
SA inhibits TNF-α-induced chondrocyte inflammatory factor expression and extracellular matrix degeneration. Chondrocytes were stimulated with TNF-α and then treated with or without 10 μM SA for 48 h. **a** Relative mRNA expression levels of IL1β, IL-6, MMP13, COL2A1 and ACAN were detected by reverse transcription-quantitative polymerase chain reaction (*n* = 3, one-way ANOVA). **b** Expression of active MMP13 protein was reduced by the chondrocytes when stimulated with SA (*n* = 3, one-way ANOVA). **c** WB analysis for detecting the cartilage matrix markers MMP13, ACAN and COL2A1 in different groups (i). Quantitative analysis of MMP13 (ii), ACAN (iii), and COL2A1 (iv) at the protein level. GAPDH was used as a reference protein (*n* = 3, one-way ANOVA). The data are expressed as the mean ± SD, *p < 0.05, **p < 0.01, ***p < 0.001, and ns not significant.

We first explored the effects of SA on TNF-α-induced chondrocyte inflammatory factors and matrix gene expression. As shown in Fig. 2a, TNF-α dramatically increased interleukin-1β (IL-1β), interleukin-6 (IL-6), and matrix metalloproteinase 13 (MMP13) gene expression and inhibited Col2a1 and ACAN gene expression in chondrocytes compared with that of the control group. However, these effects could be diminished by 48 h of treatment with 10 μM SA in vitro. Moreover, we found that TNF-α induced chondrocyte matrix degeneration by elevating the expression and secretion of MMP13, which was reversed by SA at the protein level (Fig. 2b-c). In addition, we found that SA could rescue TNF-α-induced cartilage matrix degradation by detecting the expression of Col2a1 and ACAN at the protein level (Fig. 2ci). Quantitative analysis showed the same trend (Fig. 2cii-iv). These results indicated that SA inhibits TNF-α-induced chondrocyte degeneration in vitro.

### SA ameliorates cartilage degeneration by enhancing chondrocyte proliferation and inhibiting chondrocyte apoptosis

**Fig. 3:**
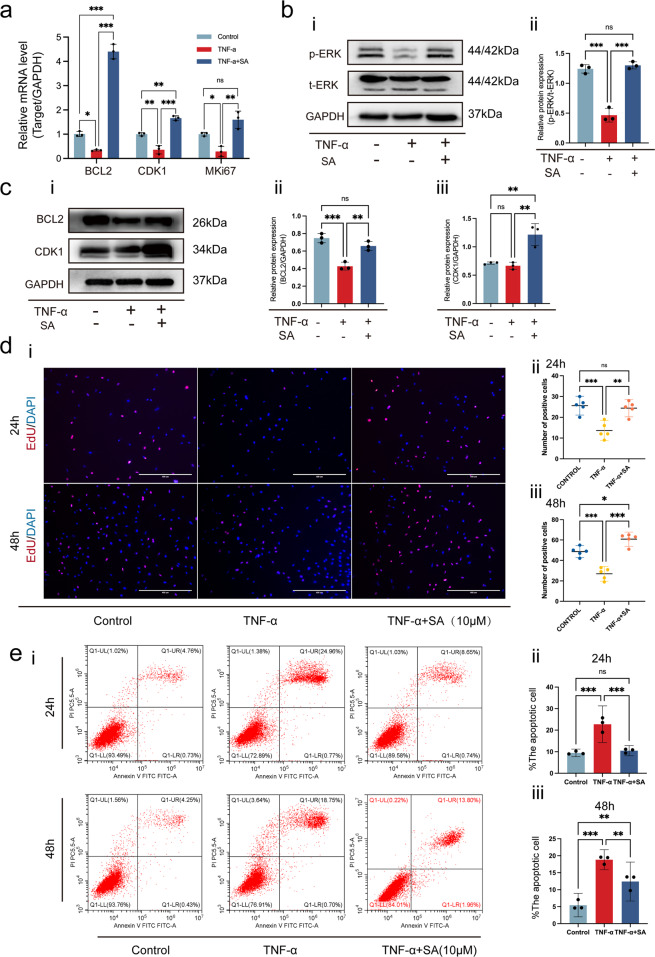
SA rescues TNF-α-induced inhibition of chondrocyte proliferation and TNF-α-induced promotion of chondrocyte apoptosis. **a** SA rescued TNF-α-induced inhibition of cell cycle marker expression at the mRNA level. Chondrocytes were stimulated with TNF-α and then treated with or without 10 μM SA for 48 h, and the relative mRNA expression levels of BCL2, CKD1, and MKi67 were detected (*n* = 3, one-way ANOVA). **b** Western blot analysis for detecting t-ERK and p-ERK in different groups treated with SA for 48 h (**a**). Quantitative analysis of the ratio of p-ERK/t-ERK. GAPDH was used as a reference protein (**b**) (*n* = 3, one-way ANOVA). **c** WB for detecting the proliferation markers BCL2 and CDK1 in different groups 48 hours after treatment at the protein level. Quantitative analysis of BCL2 (ii) and CDK1 (iii) at the protein level. GAPDH was used as a reference protein (*n* = 3, one-way ANOVA). **d** EdU assays for detecting DNA synthesis indicating cell proliferation with 24 h and 48 h treatment (i), scale bar 400 μm. Percentage of EdU (red)-positive staining at 24 h (ii) and 48 h (**c**) (*n* = 5, random fields, one-way ANOVA). **e** Flow cytometry for cell apoptosis analysis of chondrocytes in each treatment group at 24 h and 48 h (i). Percentage of apoptotic cells in each treatment group at 24 h (ii) and 48 h (**c**) (*n* = 3, one-way ANOVA). The data are expressed as the mean ± SD, *p < 0.05, **p < 0.01, ***p < 0.001, and ns not significant.

Next, we investigated the effects of SA on TNF-α-mediated chondrocyte apoptosis and proliferation. We found that TNF-α significantly inhibited the expression of the antiapoptotic gene BCL-2 and the cell cycle genes CDK1 and MKi67 in chondrocytes. However, when the chondrocytes were treated with 10 μM SA, these inhibitory effects were eliminated (Fig. 3a). Regarding the phosphorylation of ERK, we found that TNF-α did not influence total ERK (t-ERK) expression, but it significantly inhibited p-ERK expression. SA blocked the downregulation of p-ERK and elevated the ratio of p-ERK and t-ERK in comparison to the TNF-α treatment (Fig. 3b). In addition, we determined the effects of SA on TNF-α-mediated inhibition of BCL2 and CDK1 expression at the protein level. We found that SA eliminated TNF-α-induced inhibition of BCL2 (Fig. 3ci), but TNF-α did not inhibit the expression of CDK1 at the protein level, and SA promoted the expression of CDK1 in the presence of TNF-α (Fig. 3ci). Quantitative analysis showed the same trend (Fig. 3cii–iii). These results suggested that SA eliminated TNF-α-mediated inhibition of cell cycle-related marker expression.

Furthermore, cell proliferation by EdU staining showed that SA could rescue TNF-α-induced inhibition of DNA duplication in a dose-dependent manner at both 24 h and 48 h (Fig. 3di). Quantitative analysis showed the same trend (Fig. 3dii–iii). Cell apoptosis and quantitative analysis also confirmed that SA eliminated the TNF-α-induced promotion of cell apoptosis at both 24 h and 48 h (Fig. 3e).

### SA inhibits IRE1α-IκBα-p65 signaling-mediated ER stress by directly binding to IRE1α

**Fig. 4:**
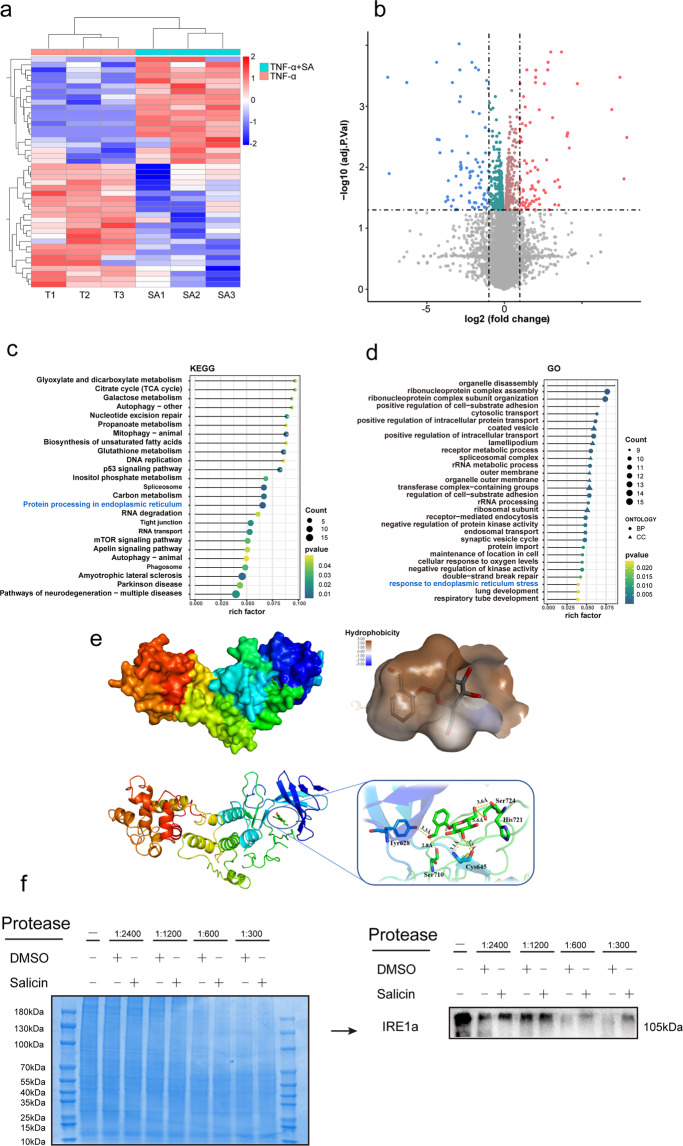
RNA-seq and bioinformatics analysis for estimating molecular targets of SA. **a** Heatmap for global gene expression with group clusters (*n* = 3). **b** Volcano map of DEGs in the SA group vs. the control group (upregulation: 75 genes and downregulation: 32 genes). DEGs with FC (fold change) ≥ 2 were accepted as positive DEGs. **c** Pathway enrichment bubble map based on the KEGG enrichment analysis. A rich factor indicates a higher degree of enrichment, a larger *p* value (−log10) indicates a higher statistical significance, and a larger bubble indicates a higher degree of enrichment. **d** GO enrichment of those DEGs. A rich factor indicates a higher degree of enrichment, a larger *p* value (−log10) indicates a higher statistical significance, and a larger bubble indicates a higher degree of enrichment, BP: biological process, CC: cellular component. **e** Molecular docking for estimating the binding site of IRE1α and SA. The three-dimensional (3D) structures of IRE1α and SA were subjected to molecular docking, and the binding site was obtained according to the software scoring system. Upper left panel: SA was located in the cavity formed by the IRE1α 3D structure, upper right panel: magnification of the cavity; lower left panel: binding site of SA and IRE1α, lower left panel: magnification showing the hydrogen bonds (yellow dot line) formed between SA and IRE1α. **f** DARTS assay for confirmation of IRE1α and SA binding. Protease was used to digest IRE1α protein. With the addition of SA, the digestion of IRE1α was significantly blocked at each concentration of protease compared with the DMSO group.

RNA-seq was performed in chondrocytes cultured with or without SA under TNF-α stimulation. As shown in Fig. 4a, b, for differentially expressed genes (DEGs), we found that 94 DEGs were upregulated, and 96 DEGs were downregulated. Then, DEGs were subjected to GO and KEGG signaling pathway enrichment analysis (Fig. 4c, d). Through GO analysis, we found that DEGs were highly enriched in biological processes (BPs) such as cellular responses to ER stress and oxygen levels. In addition, KEGG signaling pathway enrichment analysis showed that protein processing in the ER (Fig. 4c, d marked with blue) was one of the most highly enriched signaling pathways.

Three main pathways mediated the transduction of ER stress. As shown in Supplementary Fig. 2, SA did not influence TNF-α induced PERK phosphorylation and the expression of ATF6. However, SA dramatically rescued TNF-α induced IRE1-α phosphorylation. Therefore, we analyzed the potential combination of SA and IRE1α.

Three-dimensional (3D) structures of SA and IRE1α were subjected to molecular docking. The score of SA docking with IRE1α was −8.567 kcal/mol, and the theoretical binding mode is shown in Fig. 4e. The compound and the amino acid residue TYR628 formed a hydrogen bond with a bond length of 3.3 Å; the amino acid residue CYS645 formed a hydrogen bond with a bond length of 3.2 Å; and the amino acid residue SER724 (phosphorylation site) formed a hydrogen bond with a bond length of 3.6 Å. These interactions guaranteed the stability of the combination of IRE1α and SA. Furthermore, through the DARTS assay, we found that SA protected IRE1α from dosage-dependent protease digestion compared with the control (Fig. 4f), which indicates the direct combination of SA and IRE1α protein.

**Fig. 5:**
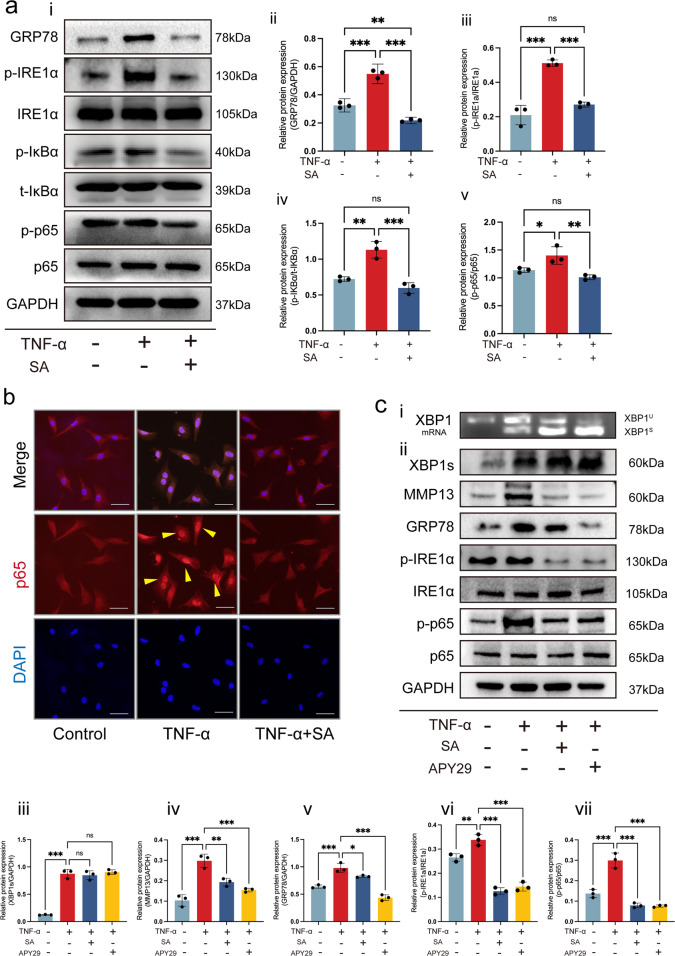
SA regulates IRE1α-mediated ER stress by IRE1α-IκBα-p65 signaling. **a** WB analysis for detecting IRE1α and downstream gene expression at the protein level. The ER stress-associated proteins GRP78, pIRE1α, IRE1α, p-IκBα, t-IκBα, p-p65 and p65 were detected in each group (i). Quantitative analysis of GRP78 (ii), the ratio of p-IRE1α/t-IRE1α (iii), the ratio of p-IκBα/t-IκBα (iv), and the ratio of p-p65/t-p-p65 (v) at the protein level. GAPDH was used as a reference protein (*n* = 3, one-way ANOVA). **b** P65 nuclear translocation in each treatment group. IF was used to detect p-65 in the nucleus, and DAPI was used to stain the cell nucleus; scale bar: 50 μm. **c** The IRE1α inhibitor APY29 blocked TNF-α-mediated ER stress. Chondrocytes were stimulated with TNF-α and then treated with 10 μM SA or 10 μM APY29 for 48 h. XPB1^u^ and XPB1^s^ were detected by RT-QPCR (i), and XPB1s, MMP13, GRP78, IRE1α, p-IRE1α, p-p65 and p65 were detected by WB analysis (ii). Quantitative analysis of XPB1s (iii), MMP13 (iv), GRP78 (v), the ratio of p-IRE1α/t-IRE1α (vi), and the ratio of p-p65/t-p65 (vii) at the protein level. GAPDH was used as a reference protein (*n* = 3, one-way ANOVA). The data are expressed as the mean ± SD, *p < 0.05, **p < 0.01, ***p < 0.001, and ns, not significant.

Next, we investigated the regulatory effects of SA on IRE1α-mediated ER stress. First, we detected the expression level of the ER stress marker GRP78 and found that SA inhibited TNF-α-initiated GRP78 expression by western blotting (WB; Fig. 5ai) and quantitative analysis (Fig. 5aii). Total IRE1α and p-IRE1α levels were also detected by WB. Compared to the control, TNF-α did not influence total IRE1α expression but downregulated phosphorylated IRE1α expression, which could be rescued by SA treatment (Fig. 5aiii). Then, we detected IRE1α downstream gene NF-kappa-B inhibitor alpha (IκBα) and p65 expression at the protein level. The results showed that TNF-α induced high expression of p-IκBα and p65, which could be diminished by SA treatment (Fig. 5aiv–v).

Subsequently, we explored whether SA influenced the TNF-α-induced distribution of p65 protein in chondrocytes by IF. As expected, TNF-α-induced p65 translocation from the cytoplasm into the nucleus was eliminated by SA treatment (Fig. 5b). These findings indicated that SA inhibited IRE1α-IκBα-p65 signaling-mediated ER stress by occupying the phosphorylation site of IRE1α.

Finally, the IRE1α inhibitor APY29 was utilized to confirm the function of SA. We found that the IRE1-regulated transcription factors XBP1u and XBP1s were upregulated with the stimulation of TNF-α, and SA and APY29 did not influence XBP1u or XBP1s expression at the mRNA level (Fig. 5ci) or XPB1s (Fig. 5cii) at the protein level, which indicated that SA does not regulate IRE1α-mediated ER stress through XPB1s signaling. However, APY29 dramatically inhibited TNF-α-induced ER stress marker GRP78, p-IRE1α, p-p65 and cartilage matrix degeneration marker MMP13 expression at the protein level, and SA exhibited a similar effect (Fig. 5ciii–vii).

Taken together, these results suggested that SA can directly bind to IRE1α and inhibit IRE1α phosphorylation, which further blocked IRE1α-IκBα-p65 signaling-mediated ER stress.

### SA intra-articular injection ameliorates ACLT-induced OA progression by inhibiting IRE1α-mediated ER stress

**Fig. 6:**
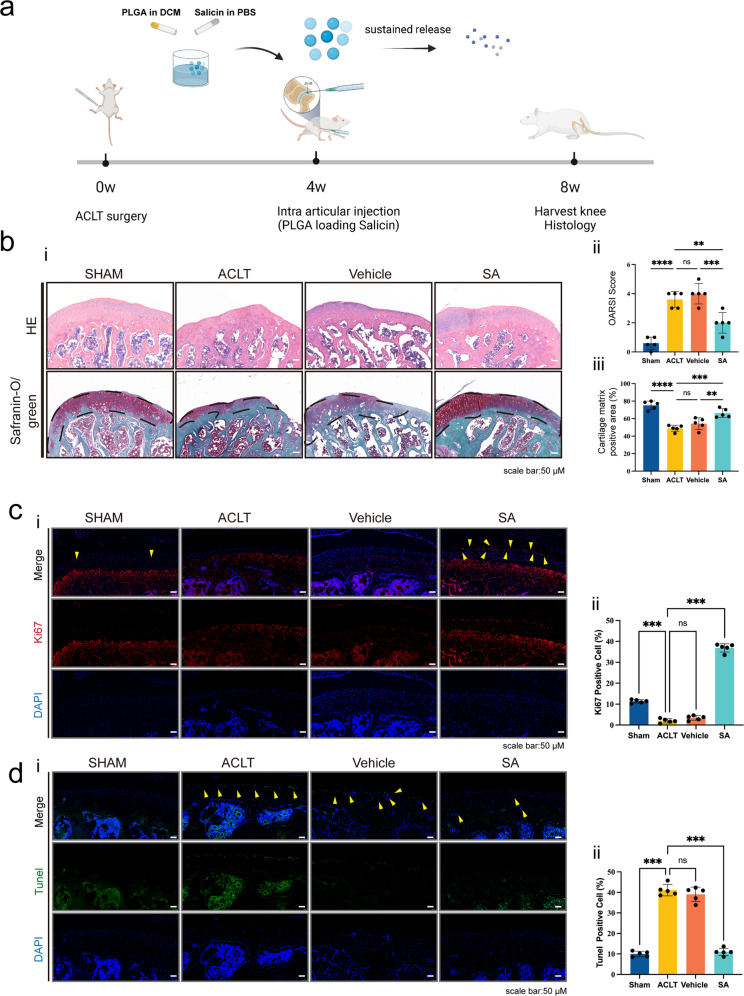
SA intra-articular injection ameliorates ACLT-induced OA progression. **a** Diagram summarizing the animal experiments. ACLT was used to construct the knee OA model. Four weeks later, intra-articular injection of SA-loaded PLGA was performed, and intra-articular injection of PLGA only was used as a control. Four weeks after treatment, rats in each group were sacrificed, and knee joints were subjected to histological analysis. **b** H&E and safranin O/green staining for each treatment group. H&E (upper panel) and safranin O/green staining (lower panel) showed that ACLT resulted in thicker cartilage and degeneration of the cartilage matrix than sham treatment, and intra-articular injection of SA-loaded PLGA ameliorated this progression compared with vehicle (PLGA only) treatment or sham treatment. OARSI scoring (ii) and cartilage matrix area quantitative analysis (iii) showed that, although OARSI scores in the ACLT, vehicle and SA groups were significantly higher than that of the sham group, the OARSI score in the SA group was significantly lower than those of the ACLT and vehicle groups (*n* = 5, one-way ANOVA). **c** Ki67 staining for detecting chondrocyte proliferation. IF staining was used to detect Ki67-positive chondrocytes in each treatment group (i). Quantitative analysis showed that SA dramatically promoted chondrocyte Ki67 expression compared with ACLT (*n* = 5, one-way ANOVA). **d** TUNEL staining for detecting chondrocyte apoptosis. TUNEL staining showed that SA treatment reversed ACLT-induced chondrocyte apoptosis (i). Quantitative analysis showed the same trend (ii) (*n* = 5, one-way ANOVA). ACLT group indicates the group with PBS injection; vehicle group indicates the group injected with only PLGA vehicle; SA group indicates the group injected with SA-loaded PLGA. Dashed lines indicate the cartilage surface. Scale bar 50 μm, data are expressed as the mean ± SD, *p < 0.05, **p < 0.01, ***p < 0.001, and ns, not significant.

A rat ACLT-induced OA model was constructed for further research. Figure 6a summarizes the protocol of the in vivo study. SA-loaded PLGA scaffolds were intraarticularly injected for controlled release of SA^27,28^. As shown in Fig. 6bi, no obvious cartilage degeneration was found in the sham group. In the ACLT and vehicle groups, cartilage cell number, cartilage matrix and cartilage thickness decreased significantly compared with those of the sham group, while the SA group showed diminished effects. Knee joint OARSI scoring (Fig. 6bii) and quantitative analysis of the articular cartilage area (Fig. 6biii) showed that SA treatment could reverse ACLT-induced cartilage degeneration.

**Fig. 7:**
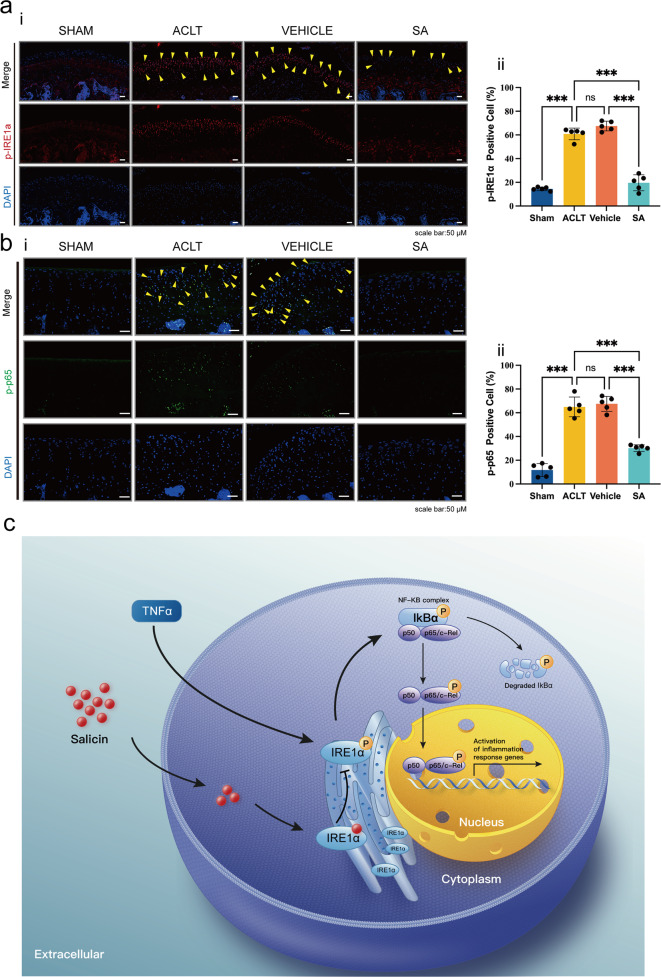
SA ameliorates ACLT-induced OA progression by inhibiting IRE1α-mediated ER stress in vivo. **a** P-IRE1α in chondrocytes in each treatment group. IF was used to detect p-IRE1α in each treatment group. p-IRE1α was highly expressed in the cytoplasm of chondrocytes in the ACLT and vehicle groups, and SA dramatically decreased the expression of p-IRE1α in the cytoplasm of chondrocytes (i). Quantitative analysis showed that ACLT-induced high expression of p-IRE1α was significantly reversed by SA treatment (ii) (*n* = 5, one-way ANOVA). **b** P-p65 nuclear translocation in each treatment group. ACLT-induced p-p65 nuclear translocation was ameliorated by SA treatment (i). Quantitative analysis of each treatment group showed the same trend (ii). Scale bar, 50 μm; the ACLT group indicates the group injected with PBS; the vehicle group indicates the group injected with only PLGA vehicle; the SA group indicates the group injected with SA-loaded PLGA. Dashed lines indicate the cartilage surface. The data are expressed as the mean ± SD, *p < 0.05, **p < 0.01, ***p < 0.001, and ns, not significant. **c** A proposed model of action. SA binding to IRE1α blocked IRE1α phosphorylation and inhibited IRE1α-mediated ER stress via IRE1α-IκBα-p65 signaling.

To further prove the mechanisms by which SA inhibits IRE1α-mediated ER stress, we performed IF. Ki67 staining and TUNEL staining were used to evaluate chondrocyte proliferation and apoptosis. The results showed decreased proliferation and increased apoptosis of chondrocytes in both the ACLT and vehicle groups compared with the sham group, while SA treatment reversed the decreased proliferation and increased apoptosis of chondrocytes (Fig. 6c, d). Then, we found that the OA-induced high phosphorylation levels of IRE1α were alleviated by SA treatment (Fig. 7a). Finally, we showed that OA-induced p-p65 nuclear translocation was inhibited by SA treatment (Fig. 7b). These results suggested that SA ameliorates ACLT-induced OA progression by inhibiting IRE1α-mediated ER stress in vivo.

In summary, our in vitro and in vivo tests showed that SA binds to IRE1α and blocks IRE1α phosphorylation, which inhibits IRE1α-mediated ER stress by IRE1α-IκBα-p65 signaling (Fig. 7c).

## Discussion

With the aging population, the morbidity of OA is increasing year by year^33^. DMOADs have been identified as potential treatments to address the increasing OA prevalence^2,34^. In this study, we found that SA not only inhibits TNF-α-induced chondrocyte inflammatory factor expression and extracellular matrix degeneration but also enhances chondrocyte proliferation and inhibits chondrocyte apoptosis. Mechanistically, we clarified that SA may regulate OA progression by binding with IRE1α and regulating IRE1α-mediated ER stress.

As the most common chemically standardized willow bark extract, SA is a nonsteroidal anti-inflammatory drug (NSAID), which may cause gastrointestinal, renal, and cardiovascular toxicity^6,34^. As reported by Schmid et al., oral ingestion of 13.97 mmol of pure SA resulted in a peak serum concentration of 800 µM, which indicated that oral ingestion of SA could lead to side effects or low concentrations of SA in the knee joint^7^. As a natural small molecular drug, SA can cross the cell membrane and bind with intracellular targets, thus avoiding the serious side effects caused by oral ingestion. By directly adding SA to the chondrocyte medium and intra-articular injection of SA-loaded PLGA, we demonstrated the anti-inflammatory, pro-proliferative, and antiapoptotic effects of SA in the progression of OA. These results indicate the advantage and efficiency of intra-articular use of SA.

Recently, in addition to the anti-inflammatory, antipyretic, antirheumatic, and antiseptic properties of SA^10^, Gao et al.^12^ found that SA prevents collagen type II degradation by inhibiting the activation of the NF-κB proinflammatory pathway. However, the underlying detailed mechanisms are still unclear. In this study, we obtained similar results by detecting cartilage matrix degeneration and inflammatory markers. Furthermore, DEGs found by RNA sequencing and KEGG enrichment analysis showed that ER stress was highly involved in the protective effects of SA on cartilage degeneration. Next, molecular docking and DARTS analysis showed how SA can occupy the phosphorylation sites of IRE1α and block IRE1α phosphorylation-mediated p65 nuclear translocation and downstream gene expression. Thus, this study helped elucidate the SA-mediated protective effects on cartilage degeneration.

The ER is a multifunctional organelle where protein folding occurs prior to transport to the extracellular surface or different intracellular sites^35–38^. Three ER transmembrane proteins mediate the unfolded protein response (UPR), including IRE1, pancreatic endoplasmic reticulum kinase (PERK), and activating transcription factor 6 (ATF6)^36,37^. These three ER transmembrane proteins regulate thousands of genes involved in controlling the function of the ER while also regulating signal transduction of ER stress^39,40^. Among them, IRE1 is the most conserved gene from yeast to humans and has two subtypes: IRE1α and IRE1β. IRE1α is commonly expressed in most cells and tissues, while IRE1β is limited to gastrointestinal epithelial cells^41^. Notably, IRE1α plays a key role in chondrocyte proliferation, ECM production, etc^42^., which indicates the regulatory function of IRE1α-mediated ER stress in chondrocyte proliferation and degeneration. Jacqueline et al.^43^ found that the two top risk factors for OA, age and obesity, were highly associated with ER stress, and resveratrol mitigated early joint degeneration by inhibiting ER stress. Benedetta et al.^44^ found that chronic ER stress decreased chondrocyte proliferation. Kung et al.^45,46^ reported that hypertrophic chondrocytes have limited potential to cope with increased ER stress, and they also found that increased ER stress is sufficient to reduce chondrocyte proliferation. Rajpar et al.^47^ showed that ER stress plays a direct role in cartilage pathology. It is widely accepted that ER stress is directly associated with chondrocyte apoptosis and death^48–50^. Moreover, recent studies have confirmed that OA inflammation is related to ER stress^51–55^. In this study, we first detected the expression levels of PERK, p-PERK, IRE1α, p-IRE1α, and AFT6 after stimulation with TNF-α in the presence of SA and found that SA did not influence TNF-α-induced PERK phosphorylation or ATF6 expression. However, SA dramatically rescued TNF-α-induced IRE1-α phosphorylation. Furthermore, we showed that by directly binding with IRE1α, SA inhibited IRE1α-mediated ER stress and subsequently promoted chondrocyte proliferation, decreased inflammatory factor expression, and inhibited chondrocyte apoptosis through both in vitro and in vivo tests.

Recently, ER stress regulators were identified as new drug targets for several diseases^35–37,56^. During the progression of OA, chondrocytes are responsible for the biogenesis and maintenance of cartilage ECM. Several cellular stresses, including hypoxia, oxidative stress, nutrient deprivation, aging, injury, etc., can cause excessive unfolding or misfolded proteins on the ER and trigger ER stress^43,50^. Huang et al.^42^ reported that IRE1α regulates chondrocyte apoptosis by activating NF-κB signaling. Ye et al.^57^ reported that phosphorylated IRE1α activates NF-κB signaling by releasing IκBα. Released NF-κB dimers translocate to the nucleus and bind κB sites in the promoters or enhancers of target genes^58^. However, IRE1α can also activate RNase to splice XBP1 mRNA and produce the homeostatic transcription factor XBP1s, which mainly participates in ER chaperone expression, lipid biosynthesis, hexosamine biosynthesis and proinflammatory cytokine expression^16,40,59^. We showed that instead of influencing the expression of XBP1u and XPB1s at the mRNA level or influencing the expression of XPB1s at the protein level, SA regulated ER stress by regulating p-IRE1α and p-IκBα and activating p-p65 nuclear translocation. Furthermore, molecular docking and DARTS analysis showed that SA can bind the phosphorylation site of IRE1α and block IRE1α phosphorylation, which is followed by the decreased expression of p-κBα and p-p65 nuclear translocation. Therefore, as one of the key ER stress regulators, IRE1α is a potential drug target for OA treatment.

As a whole joint disease, OA, especially late-stage OA, is characterized by dramatic synovium, cartilage, and subchondral bone pathologies^1,60,61^. However, when early-stage OA featured cartilage pathology^62,63^, we found that topical use of SA promoted chondrocyte proliferation and inhibited cartilage matrix degeneration. Therefore, SA shows potential as an effective drug for modifying early-stage OA. More studies that focus on the effect of SA on other cell types, such as synoviocytes and immune cells, are needed and will contribute to the further clinical translation of SA.

This study demonstrated that SA directly binds to IRE1α and blocks IRE1α phosphorylation, which inhibits IκBα phosphorylation and p65 nuclear translocation. Subsequently, these events inhibit chondrocyte apoptosis, promote chondrocyte proliferation, and decrease the expression of inflammatory factors. Thus, SA is a potential drug for the clinical modification of OA progression by intra-articular injection.

## Supplementary information

Supplementary materials
