# Supplementary material for: The natural product salicin alleviates osteoarthritis progression by binding to IRE1α and inhibiting endoplasmic reticulum stress through the IRE1α-IκBα-p65 signaling pathway
Source: Exp Mol Med. 2022 Nov 10;54(11):1927–39. doi: 10.1038/s12276-022-00879-w (PMC9722708; doi:10.1038/s12276-022-00879-w)
Supplement: Supplementary file 1 — Supplementary materials [file 12276_2022_879_MOESM1_ESM.pdf]

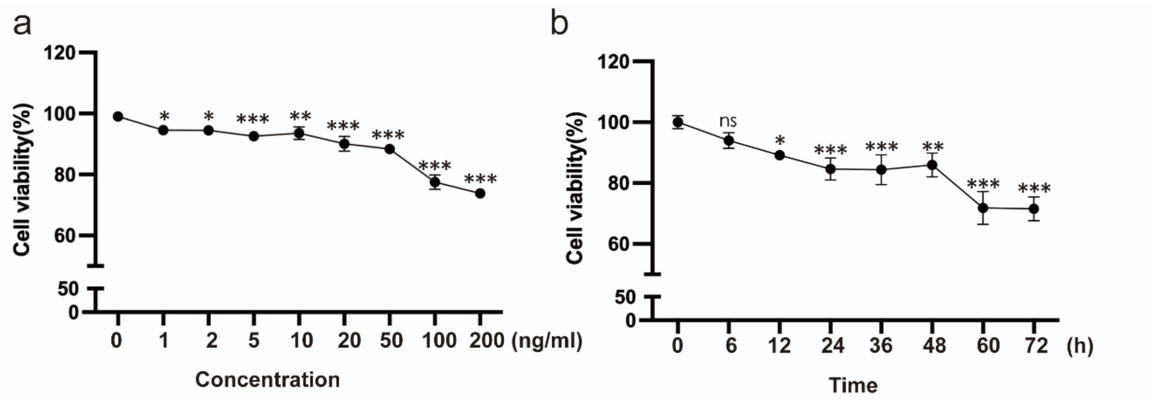

Supplementary Fig. 1: Cytotoxicity of TNF-α on primary articular chondrocytes. (a) Chondrocytes were treated with TNF-α in gradient concentration (0-200ng/ml) for 48h and subjected to CCK-8 analysis. Cell viability was calculated from OD value compared with 0 ng/ml group (Control group) (n=3, one-way ANOVA). (b) Chondrocytes were treated with TNF-α (10ng/ml) from different time points (0, 6, 12, 24, 36, 48, 60, 72h), compared with 0h group (Control group), (n=3, one-way ANOVA). The data are expressed as mean ± SD, *$p < 0.05$, **$p < 0.01$, ***$p < 0.001$, and ns, not significant. *Compared with Control group.

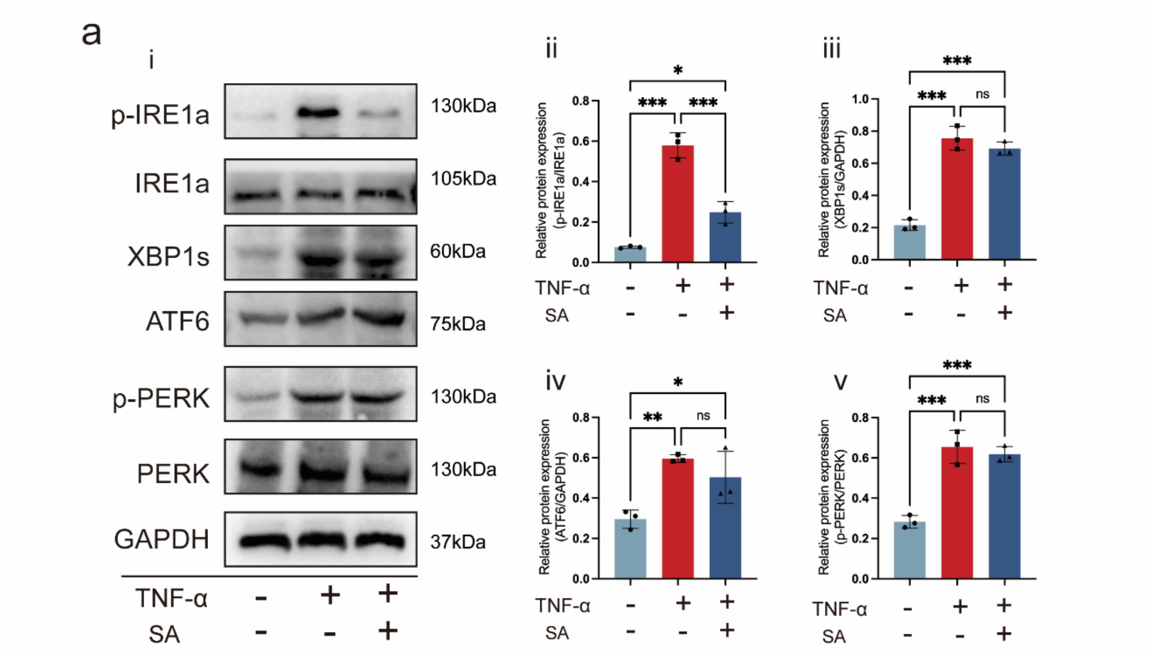

Supplementary Fig. 2: SA regulates endoplasmic reticulum stress by inhibiting the phosphorylation of IRE1α. (a) WB analysis for detecting genes expression at protein level. ER stress associated protein pIRE1α, IRE1α, XBP1s, ATF6, p-PERK and PERK were detected in each group (i). Quantitative analysis of ratio of p-IRE1α/IRE1α(ii), XBP1s

expression (iii), ATF6 expression (iv) and ratio of p-PERK/PERK (v) at protein level, GAPDH was used as reference protein (n=3, one-way ANOVA). The data are expressed as mean ±SD, *$p < 0.05$, **$p < 0.01$, ***$p < 0.001$, and ns, not significant. *Compared with Control group.
